# Patterns and causes of liver involvement in acute dengue infection

**DOI:** 10.1186/s12879-016-1656-2

**Published:** 2016-07-08

**Authors:** Samitha Fernando, Ananda Wijewickrama, Laksiri Gomes, Chameera T. Punchihewa, S. D. P. Madusanka, Harsha Dissanayake, Chandima Jeewandara, Hemantha Peiris, Graham S. Ogg, Gathsaurie Neelika Malavige

**Affiliations:** Department of Microbiology, Centre for Dengue Research, Faculty of Medical Sciences, University of Sri Jayewardenepura, Nugegoda, 10250 Sri Lanka; Department of Anatomy, Faculty of Medical Sciences, University of Sri Jayewardenepura, Nugegoda, 10250 Sri Lanka; Department of Family Medicine, Faculty of Medical Sciences, University of Sri Jayewardenepura, Nugegoda, 10250 Sri Lanka; Department of Biochemistry, Faculty of Medical Sciences, University of Sri Jayewardenepura, Nugegoda, 10250 Sri Lanka; Infectious Diseases Hospital, Angoda, Sri Lanka; MRC Human Immunology Unit, Weatherall Institute of Molecular Medicine, Oxford NIHR Biomedical Research Centre and University of Oxford, Oxford, OX3 9DS UK

**Keywords:** Dengue, Liver injury, Transaminases, Viral loads, Immune mediated, Fluid leakage

## Abstract

**Background:**

Liver involvement in acute dengue infection is frequently observed and sometimes leads to acute liver failure, with fatal outcomes. Many factors are thought to contribute to liver dysfunction, including hypoxic injury due to decreased perfusion, direct damage by the virus and immune mediated injury. In this study, we sought to identify the pattern in the change in liver enzymes throughout the illness and its association with the degree of viraemia, onset and extent of plasma leakage and inflammatory mediators.

**Methods:**

Serial daily blood samples were obtained from 55 adult patients with acute dengue from the time of admission to discharge and the liver function tests, viral loads and cytokines were assessed. The onset and extent of fluid leakage was measured by daily ultrasound examinations and all clinical and laboratory features were serially recorded.

**Results:**

Aspartate transaminase (AST), alanine transaminase (ALT) and gamma glutamyl transferase (GGT) levels were elevated in patients with dengue infection throughout the illness. The highest AST levels were seen on day 6 of illness and both AST and GGT levels were significantly higher in patients with severe dengue (SD), when compared to those with non-severe dengue (NSD) on day 5 and 6 of illness. Three patients with SD had AST and ALT values of >1000/IU in the absence of any fluid leakage or a rise in the haematocrit (≥20 %). The peak of the AST levels and the lowest serum albumin levels were seen 24 h before the maximum fluid leakage and 24 h after the peak in viraemia. Both serum IL-10 and IL-17 levels were elevated during early illness and were significantly higher in those with SD when compared to NSD.

**Conclusion:**

Dengue associated liver injury appears to peak around day 6 and 7. Therefore, liver function tests done at earlier dates might not reflect the extent of liver involvement in acute infection. Since severe liver involvement can occur in the absence of fluid leakage, after the peak viraemia, and since it is associated with high IL-17 and IL-10 levels, possible immune mechanisms leading to hepatic damage should be investigated.

**Electronic supplementary material:**

The online version of this article (doi:10.1186/s12879-016-1656-2) contains supplementary material, which is available to authorized users.

## Background

Dengue viral infections are one of the most rapidly evolving vector borne infections, which now affects 125 countries, causing approximately 100 million apparent infections each year [1]. It is predicted that the transmission of dengue will be more intensified in dengue endemic countries, and due to climate change and other factors, the infection may spread to countries that are currently not significantly affected by dengue in Europe and America [1]. Currently there is no specific antiviral or any other drug that is effective in the treatment of acute dengue infection.

Infection with the DENV results in asymptomatic disease or an undifferentiated viral fever like illness in the majority of infected individuals. However, in others, it may result in dengue fever (DF), dengue haemorrhagic fever (DHF) leading to shock (DSS), or the expanded dengue syndrome [2]. Involvement of the liver leading to hepatic dysfunction is a well-recognized complication of dengue [3–5]. Dengue associated acute liver failure has a high mortality due to complications such as encephalopathy, severe bleeding, renal failure and metabolic acidosis [3, 4]. Although dengue associated acute liver failure is thought to occur due to liver injury as a result of prolonged shock, it is also known to occur in the absence of shock [4].

Varying degrees of liver involvement are seen during acute dengue infection and are thought to result from hepatocyte apoptosis directly by the virus, hypoxic damage due to impaired liver perfusion resulting from fluid leakage, oxidative stress or immune mediated injury [6–8]. Studies done in mouse models have proposed that dengue associated liver injury can be both viral induced or immune mediated. The dengue virus (DENV) has been shown to readily infect hepatocytes in mouse models, and HepG2 and Huh7 hepatoma cell lines [9–11]. In both mouse models and hepatoma cell lines, apoptosis of hepatocytes was shown to be due to both direct viral infection and also cytokine induced [12]. It has been shown that in dengue mouse models, that both IL-22 and IL-17 were associated with severe clinical disease, and particularly liver injury [13]. In addition, liver involvement was found to be significantly reduced following IL-17 receptor blockade [13]. Again in dengue mouse models, early infiltration of the liver by natural killer cells followed by T cells was observed, and was found to associate with apoptosis of hepatocytes [14]. Cytokine mediated liver damage has also been proposed as a mechanism, as high levels of IP-10 and IL-10 were found to associate with high liver transaminase levels in children with dengue [15]. Many believe that dengue associated liver dysfunction is probably due to reduced perfusion associated hypoxic injury as a consequence of a severe vascular leak [2].

However, since the frequency of liver involvement associated with dengue is increasing and since isolated liver involvement in the absence of shock and other dengue associated complications (expanded dengue syndrome) is also increasing [2], it has become important to identify the causes of liver involvement in dengue, including the kinetics in changes in liver function tests associated with acute dengue infection. Therefore, in this study, we serially studied a cohort of adult dengue patients with severe dengue (SD) and non-severe dengue (NSD), who did not develop shock during the course of their illness, and investigated the changes in liver enzymes during the natural course of the illness and determined the degree of liver damage in association with the degree of viraemia, the onset and extent of vascular leak and changes in inflammatory mediators.

## Methods

### Patients

Fifty five adult patients with acute dengue infection were recruited from the Infectious Diseases Hospital of Sri Lanka in year 2015 from January to August, following informed written consent. Those who were known to have chronic liver disease, chronic renal disease, pregnant individuals, those on a steroid dose of >40 mg/day for over 7 days and those who had been given NSAIDs for the treatment of dengue prior to admission, were excluded from the study. None of the patients were given any drugs except for the recommended dose of paracetamol. Only those whose duration from onset of illness was ≤5 days were recruited. The day in which the patient first developed fever was considered as day one of illness. As one of the main aims of this study was to determine the timing of liver injury with changes in viral load, vascular leak and increase in inflammatory mediators, the clinical features along with these parameters were recorded daily. Blood samples were obtained daily at 10 am from the day of recruitment until discharge from the hospital. As the maximum duration of illness in this cohort was 9 days, no samples were analysed beyond day 9 of illness. The number of patients assessed at each time point is given in Additional file 1: Table S1.

Clinical features such as fever, blood pressure, pulse pressure and the urine output were measured at least 4 times a day. Serial recordings of liver function tests, viral loads, the extent of fluid leakage and serum cytokine levels were done daily. In order to determine the relationship between plasma leakage and liver derangements, we carried out serial ultrasound scans of all patients to define the onset and extent of fluid leakage. Ultrasound scanning was done in the supine position to assess the peritoneal fluid in the hepatorenal angle, subdiaphragmatic position, in paracolic gutters and in the pelvis. In order to determine the fluid in the pleural cavity the patient was kept seated upright on the examination bed for 2 min for all the pleural fluid to gravitate in to costodiaphragmatic recesses and then the patient was scanned from the back of the chest with hands raised up and crossed at the back of the head. Fluid was measured at the mid and posterior axillary lines and in seated-up position. Fluid accumulation in the peritoneal cavity was assessed by a semi quantitative manner, with classification of ascites as minimal, mild, moderate, severe and massive by looking for the presence of fluid in five areas of the abdomen namely right upper quadrant (perihepatic and morrison’s pouch), left upper quadrant (perisplenic), right paracolic gutter, left paracolic gutter and pelvis.

Severity of dengue infection was classified according to the 2009 WHO guidelines [16]. Accordingly, patients who had detectable fluid in the pleural or abdominal cavities by ultrasound scanning, or who had liver transaminases above >1000/IU were grouped as having SD. Those who had dengue with warning signs or without warning signs were classified as having non severe dengue (NSD). Signs of severe dengue were characterized by the presence of cold clammy skin, along with a narrowing of pulse pressure ≤ 20 mmHg were classified as having shock as per WHO 2009 guidelines.

### Confirmation of dengue infection and dengue antibody assays

Acute dengue infection was confirmed in the serum samples using the NS1 early dengue ELISA (Panbio, Australia) and by determining viral loads (see below). In order to determine if the patients had a primary or secondary dengue infection, the commercial capture-IgM and IgG enzyme-linked immunosorbent assay (ELISA) (Panbio, Brisbane, Australia) was used. The ELISA was performed and the results were interpreted according to the manufacturers’ instructions. This ELISA assay has been validated as both sensitive and specific for primary and secondary dengue virus infections [17, 18].

### Determining viral loads in serial blood samples

As we wished to determine the changes in viral loads in patients with SD and non-SD during the course of the illness, RNA was extracted from all serial serum samples using QIAamp Viral RNA Mini Kit (Qiagen, USA) according to the manufacturer’s protocol. The RNA was reverse transcribed into cDNA in GeneAmp PCR system 9700 using High Capacity cDNA reverse transcription kit (Applied Biosystems, USA) according to the manufacturer’s instructions. Reaction conditions were 10 min at 25 °C, 120 min at 37 °C, 5 min at 85 °C and final hold at 4 °C.

Multiplex quantitative real-time PCR was performed as previously described using the CDC real time PCR assay for detection of the dengue virus [19]. This assay was modified to quantify the DENV apart from quantitative analysis. Oligonucleotide primers and a dual labeled probe for DEN 1,2,3,4 serotypes were used (Life technologies, India) based on published sequences [19]. The probe was dual-labeled with the probe and the QSY quencher. The DENV serotype specific primers were labeled as follows: DENV-1 with JUN, DENV-2 with ABY, DENV-3 with FAM, DENV-4 with VIC. The reactions consisted of 20 μl volumes and contained the following reagents, TaqMan multiplex master mix (containing mustag dye), 900 nM of each primer, 250 nM of each probe, 2 μl of cDNA and nuclease free water (Applied Biosystems, USA). The reaction was performed in an Applied Biosystems7500, 96-well plate detection system. Following initial denaturation for 20 s at 95 °C, the reaction was carried out for 40 cycles of 3 s at 95 °C and 30 s at 60 °C. The threshold cycle value (Ct) for each reaction was determined by manually setting the threshold limit. Viral quantification (PFU/ml) of unknown samples was performed using the standard curve.

### Generating standard curves for quantification of the DENV

For generation of standard curves the four DENVs were grown in C6/36 cell lines supplemented with L15 media at 28 ° C. The virus supernatants were harvested at day 7 following infection and were immediately used to infect vero81 cell lines to determine the infective virus particles by plaque assays. The plaque assay was carried out on vero81 monolayer and the virus culture supernatants were serially diluted and inoculated in triplicate. Undiluted sample from the virus culture supernatant was used as the positive control and culture media as the negative control. After 5 days of incubation at 37 °C in 5 % CO2 incubator, the plaques were developed and counted. Virus concentration was calculated as PFU/ml. Following quantification of the viruses, the standard curves were generated as serial dilutions from 10^6^ to 10^1^ of pfu/ml of each virus serotype. In order to quantify the virus in clinical samples the unknowns were compared to known values in the standard curves of each virus and the viral loads expressed as pfu/ml (Additional file 2: Figure S1).

### Assays for determining the liver profile

Assessment of the liver profile was done on all serial blood samples on a daily basis, obtained from the time of recruitment to discharge from hospital. Liver function tests to assess liver cell damage: aspartate transaminase (AST), alanine transaminase (ALT); tests to assess synthetic functions: serum albumin; tests to assess conjugative functions: conjugated and unconjugated bilirubin and tests to assess intrahepatic and hepatobiliary cholestasis: gamma glutamyl transferase (GGT) and alkaline phosphatase (ALP) were done using automated biochemical analyzer (Thermo scientific clinical chemistry analyzer-Indiko, United States).

### Quantitative ELISA for detection of cytokines

IL-10 and IL-17 levels were determined in serial serum samples, which were collected daily from the time of admission to discharge, using the human IL-10 enzyme linked immunosorbent assay (ELISA) PRO kit (Mabtech, Sweden) and LEGEND MAX™ Human IL-17A ELISA Kit (BioLegend, USA). The ELISAs were performed and results were interpreted according to the manufacturer’s instructions.

### Statistical analysis

Statistical analysis was performed using Graph PRISM version 6. Differences in the serial values of liver enzymes, viral loads and cytokine values in patients with NSD and SD were done using multiple unpaired t tests. Corrections for multiple comparisons were done using Holm-Sidak method and the statistical significant value was set at 0.05 (alpha). The association between the extent of liver injury and clinical parameters, viral loads and inflammatory mediators was done using Spearman correlation.

## Results

Based on the WHO 2009 guidelines, 33 patients had NSD and 22 had SD. None of the patients developed shock [16]. Three patients who developed SD had diabetes and one had hypertension while 2 patients with NSD had diabetes and one had hypertension. The clinical features of the 33 patients (mean age 33.60, SD ± 13.26) with NSD and 22 patients with SD (mean age 30.36, SD ± 14.23) are shown in Table 1. Of the 22 patients with SD, 3 of them did not have any evidence of fluid leakage but were classified as having SD as their liver transaminases were elevated >1000/IU. 10 patients with SD were given dextran boluses and 4 of them were given blood transfusions, although none of them developed shock. The haematological and biochemical parameters of these patients are shown in Tables 2 and 3. Although 5 patients with NSD developed bleeding manifestations, these were mild bleeding manifestations and as the patients did not have any evidence of fluid leakage, by ultrasound scanning or by a rise in the haematocrit, they were categorized as having NSD.

**Table 1:** Clinical characteristics of patients with SD and NSD

Clinical findings	NSD (*n* = 33)	SD (*n* = 22)
Duration of fever		
3–5 days	25 (75.75 %)	15 (68.18 %)
6–8 days	8 (24.24 %)	6 (27.27 %)
>8 days	0	1 (4.54 %)
Vomiting	9 (27.27 %)	11 (50 %)
Abdominal pain	12 (36.36 %)	16 (72.72 %)
Hepatomegaly	5 (15.15 %)	15 (68.18 %)
Bleeding manifestations	5 (15.15 %)	7 (31.81 %)
Pleural effusion	0	15 (68.18 %)
Ascites	0	19 (86.36 %)
Shock	0	0

**Table 2:** Haematological changes in patients with SD and NSD

Laboratory features	NSD (*n* = 33)	SD (*n* = 22)
Lowest platelet count		
<20,000 cells/mm^3^	1 (3.03 %)	15 (68.18 %)
20,000 to 50,000	12 (36.36 %)	7 (31.81 %)
50,000–100,000	15 (45.45 %)	0
>100,000	15 (45.45 %)	0
Lowest Lymphocyte count		
<750	13 (39.39 %)	12 (54.54 %)
750–1500	19 (57.57 %)	9 (40.90 %)
>1500	1 (3.03 %)	0

**Table 3:** Biochemical findings in patients with SD and NSD

Biochemical findings	NSD (*n* = 33)	SD (*n* = 22)
AST (IU/L)		
<40	5 (15.15 %)	0
40–160	15 (45.45 %)	6 (27.27 %)
>160	13 (39.39 %)	16 (72.72 %)
ALT (IU/L)		
<40	7 (21.21 %)	3 (13.63 %)
40–160	23 (69.96 %)	11 (50 %)
>160	3 (9.09 %)	8 (36.36 %)
Albumin (g/L)		
<35	18 (54.54 %)	22 (100 %)
≥35	14 (42.42 %)	0
Total protein (g/L)		
<64	21 (63.63 %)	21 (95.45 %)
≥64	12 (36.36 %)	1 (4.54 %)
Alkaline phosphatase (IU/L)		
<128	31 (93.93 %)	18 (81.81 %)
≥128	2 (6.06 %)	4 (18.18 %)
Total bilirubin (μmol/L)		
<20	31 (96.87 %)	21 (95.45 %)
>20	1 (3.12 %)	1 (4.54 %)
Gamma glutamyl transferase (u/L)		
normal value for sex	9 (27.27)	2 (9.09 %)
>normal value for sex (female ≤ 38 and male ≤55)	24 (72.72 %)	20 (90.9 %)

### Patterns of changes in liver transaminase levels during the course of the illness

**Fig. 1:**
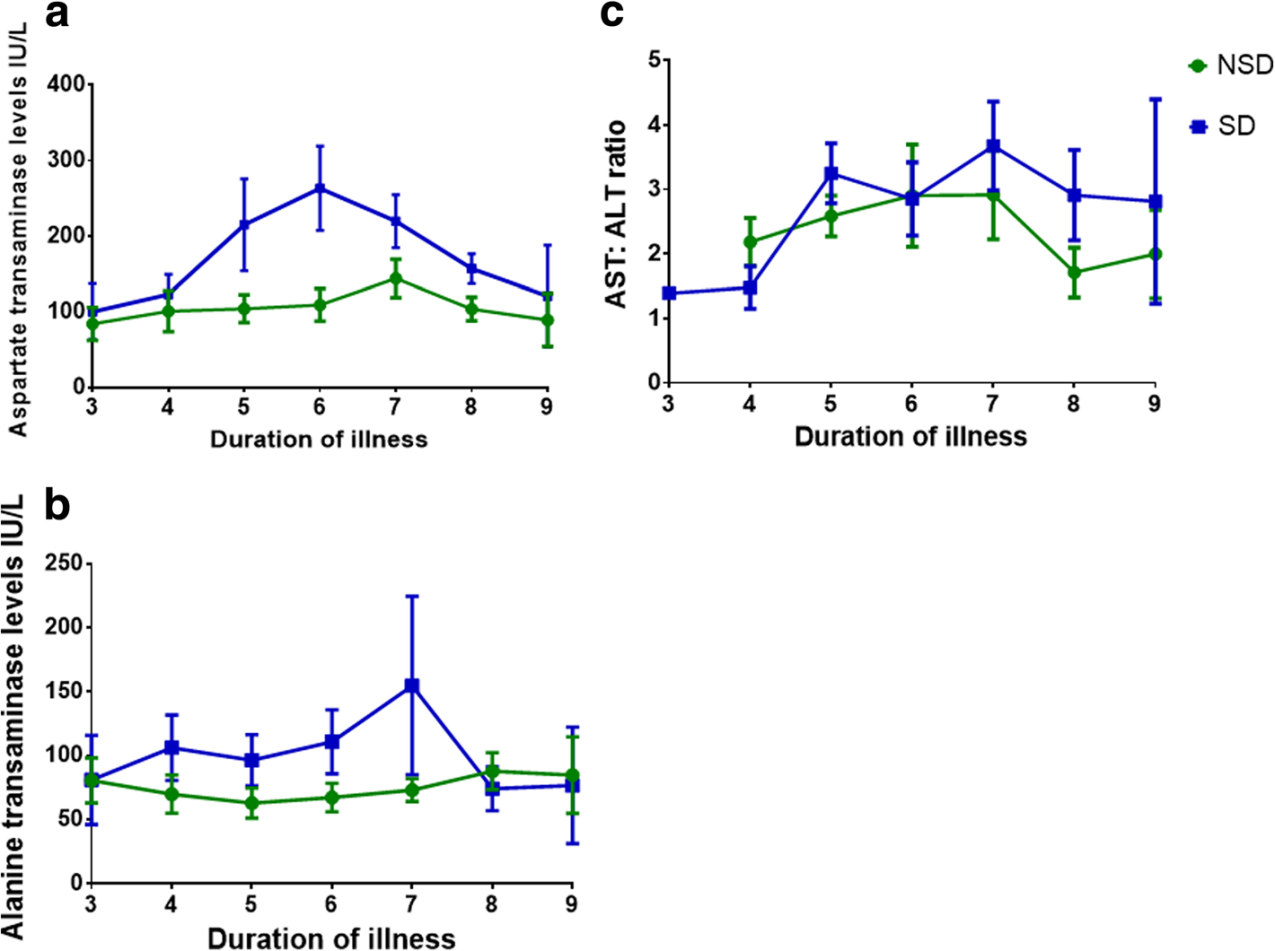
Changes in serum transaminase levels throughout the course of illness in patients with acute dengue infection. **a**: Changes in serum aspartate transaminase levels (AST) in patients with SD (*n* = 22) and NSD (*n* = 33). The lines indicate the means and standard error of mean (SEM). **b**: Changes in serum alanine transaminase levels (ALT) in patients with SD (*n* = 22) and NSD (*n* = 33). The lines indicate the means and standard error of mean (SEM). **c**: Changes in AST and ALT ratios in patients with SD (*n* = 22) and NSD (*n* = 33). The lines indicate the means and standard error of mean (SEM)

As elevation of liver transaminases commonly occurs during dengue infections, we sought to investigate the kinetics of liver transaminases in acute dengue infection and the relationship in the rise in these enzymes with viraemia, onset of fluid leakage and changes in inflammatory mediators. Both AST and ALT levels rose during the course of illness and AST peaked at day 6 of illness, while ALT peaked at day 7. After peaking at day 6 and 7 the AST and ALT levels rapidly declined (Fig. 1a and b). The AST levels were much higher in patients with SD than in those with NSD with the differences most prominent on day 5 (*p* = 0.04) and day 6 (*p* = 0.006) (Fig. 1a). The mean AST levels on day 6 (mean 263.44, SD ± 34.1 IU) were more than two fold when compared to the mean AST values on day 4 (mean 123.4, SD ± 41.8 IU) in patients with SD. The differences in ALT levels in patients with SD and NSD were not prominent (Fig. 1b). AST: ALT ratio has been considered as a better indicator of the type of liver injury than changes in AST or ALT alone [20, 21]. We found that the AST: ALT ratio was >2 in patients with SD and NSD, although the ratios were higher in those with DHF (Fig. 1c). As expected the AST levels positively correlated with other indicators of liver disease such as ALT (Spearmans correlation = 0.61, *p* < 0.0001), GGT (Spearmans correlation = 0.57, *p* < 0.0001), total bilirubin (Spearmans correlation =0.27, *p* < 0.0001) and also with IL-10 (Spearmans correlation = 0.15, *p* = 0.04).

### Changes in serum gamma glutamyl transferase (GGT) levels and alkaline phosphatase (ALP) levels

**Fig. 2:**
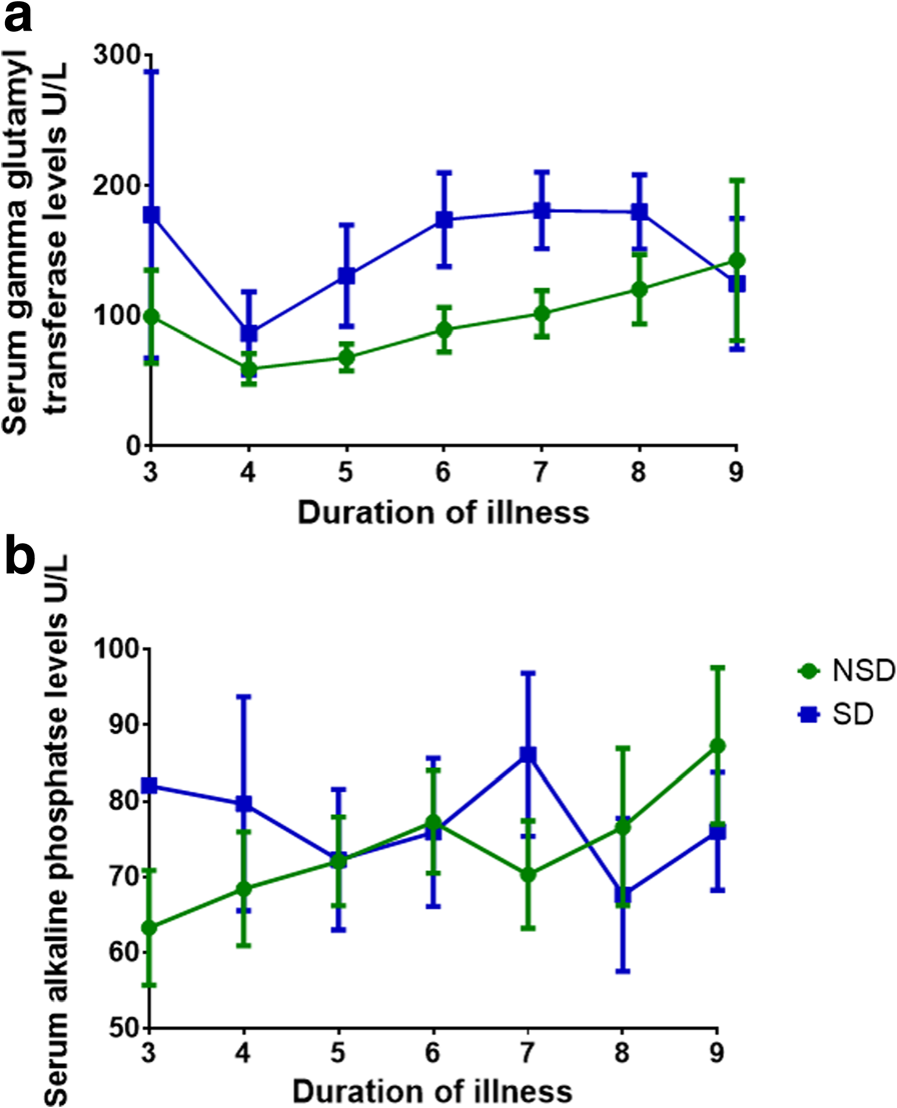
Changes in serum GGT and ALP levels throughout the course of illness in patients with acute dengue infection. **a**: Changes in serum GGT levels in patients with SD (*n* = 22) and NSD (*n* = 33). The lines indicate the means and standard error of mean (SEM). **b**: Changes in serum ALP levels in patients with SD (*n* = 22) and NSD (*n* = 33). The lines indicate the means and standard error of mean (SEM)

Although GGT is not specific to the liver, it has widely been used as a test to assess the liver profile. High levels are found with cholestasis, excessive alcohol intake, pancreatitis, diabetes and many other conditions [22]. Since it is also known to be elevated in oxidative stress [22], and since oxidative stress is thought to play a role in the pathogenesis of dengue [8, 23], we sought to investigate the changes of GGT in patients with acute dengue. We found that GGT levels were elevated throughout the course of infection in patients with both SD and NSD (Fig. 2a). The levels were significantly higher on day 6 (*p* = 0.02) and day 7 (*p* = 0.03) in patients with SD when compared to those with NSD. Twenty (90.9 %) of the patients with SD (12 males and 8 females) and 24 (72.7 %) of patients with NSD (15 males and 9 females) had elevated GGT levels.

ALP is not a liver specific enzyme and is found in many tissues such as bone, intestine and placenta apart from the liver [24]. ALP is generally elevated in hepatobiliary diseases that result in cholestatis [24]. Although 2 patients with NSD and 4 patients with SD had a mild elevation of ALP levels, all other patients had normal ALP levels throughout the course of illness, which suggests that cholestasis is unlikely to occur in dengue induced liver disease (Fig. 2b).

### Changes in bilirubin, serum albumin, plasma leakage and viraemia during the course of acute dengue

Elevation of serum bilirubin occurs due to defects in cholestasis and biliary effects. However, in acute liver disease, it is considered to be a more sensitive marker than ALT in determining the extent of liver injury [25, 26]. However, we found that the total serum bilirubin levels were only marginally high in one patient with SD and one with NSD. These patients also had normal conjugated and unconjugated bilirubin levels, further supporting that cholestatis or biliary stasis, did not significantly occur in dengue associated liver disease.

**Fig. 3:**
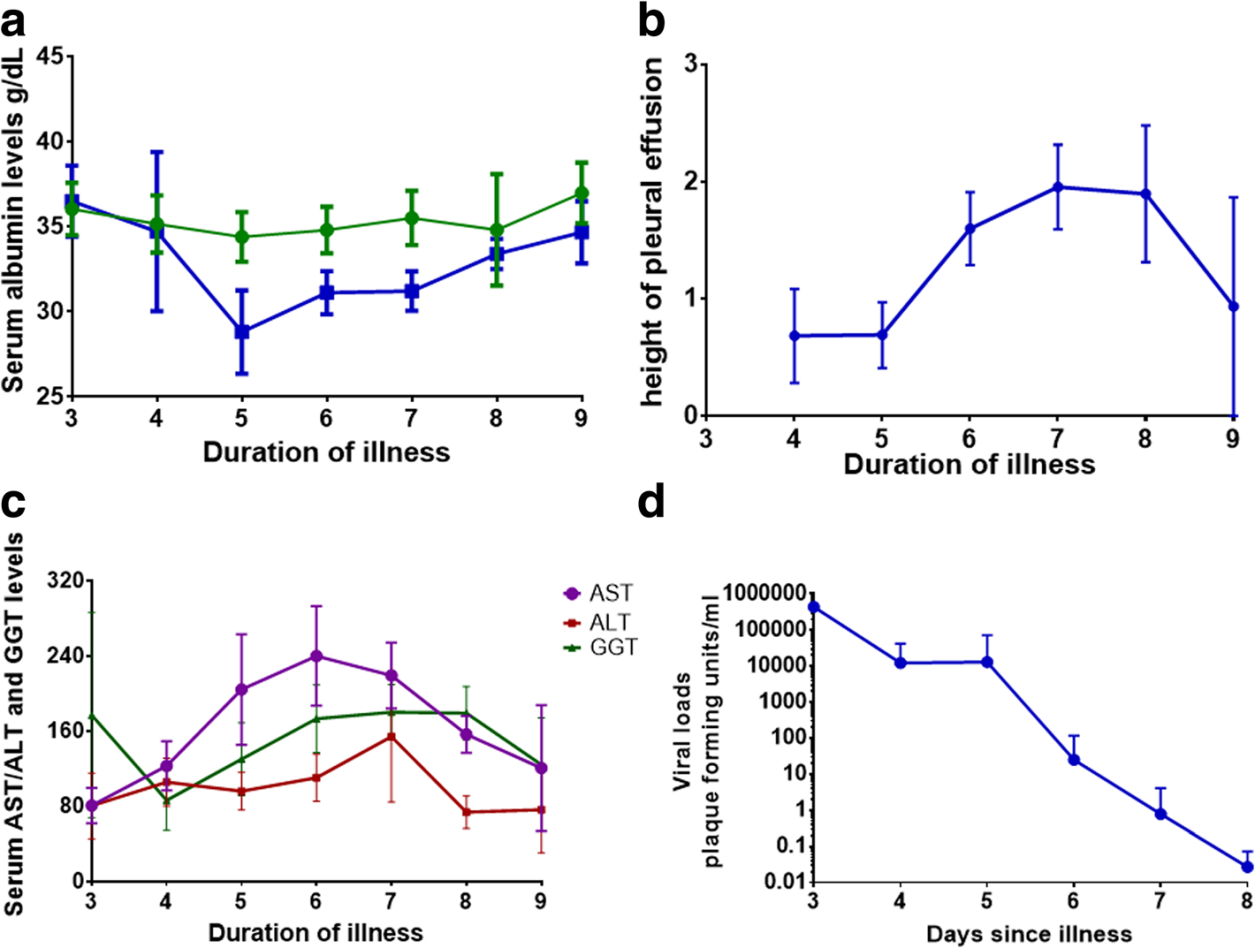
Association of liver damage with extent of fluid leakage and viraemia. **a**: Changes in serum albumin levels in patients with SD (*n* = 22) and NSD (*n* = 33). The lines indicate the means and standard error of mean (SEM). **b**: Extent of pleural effusion in patients with SD (*n* = 15, as 3 patients with SD did not have any leakage). The values indicate the height of pleural effusion in cm, over the course of illness. **c**: Changes in serum AST, ALT and GGT levels in patients with SD (*n* = 22). **d**: Changes in viral loads in patients with SD (*n* = 22)

Low serum albumin levels have been associated with severe dengue and were more frequent among those who had fatal dengue [27, 28]. As expected the serum albumin levels were significantly lower in patients with SD, especially during day 5 (*p* = 0.04), day 6 (*p* = 0.06) and day 7 (*p* = 0.04), while the values were normal in those with NSD (Fig. 3a). Interestingly, the lowest serum albumin levels were seen 2 days before the maximum peak in the fluid leakage (Fig. 3b). It is generally believed that liver dysfunction sometimes leading to acute liver failure is due to reduced hepatic perfusion secondary to prolonged shock [2]. However, it has been known to occur in the absence of shock [4]. Three of the patients in our cohort had severe liver impairment (liver transaminases >1000/IU) in the complete absence of any evidence of fluid leakage or a significant rise in the haematocrit. However, in those with SD, the maximum rise in AST and ALT levels occurred the day before the pleural effusions were largest (Fig. 3c). However, the rise in GGT levels mirrored the increase in the pleural effusions.

As studies done on HepG2 cell lines have shown that the DENV directly induced apoptosis of HepG2 cells [29], we proceeded to determine the relationship between the kinetics of viraemia with the changes in the patterns of liver enzymes. We found that the rise in liver transaminases and GGT occurred 24–48 h after the decline in the viraemia (Fig. 3d). Also, there was no association between the extent of viraemia and the extent of the rise in liver transaminases, GGT or a reduction in serum albumin levels. However, the viral loads inversely correlated with the lowest lymphocyte count observed in these patients (Spearman’s correlation = −0.6, *p* < 0.0001).

### Changes in IL-10 and IL-17 levels and extent of liver involvement

**Fig. 4:**
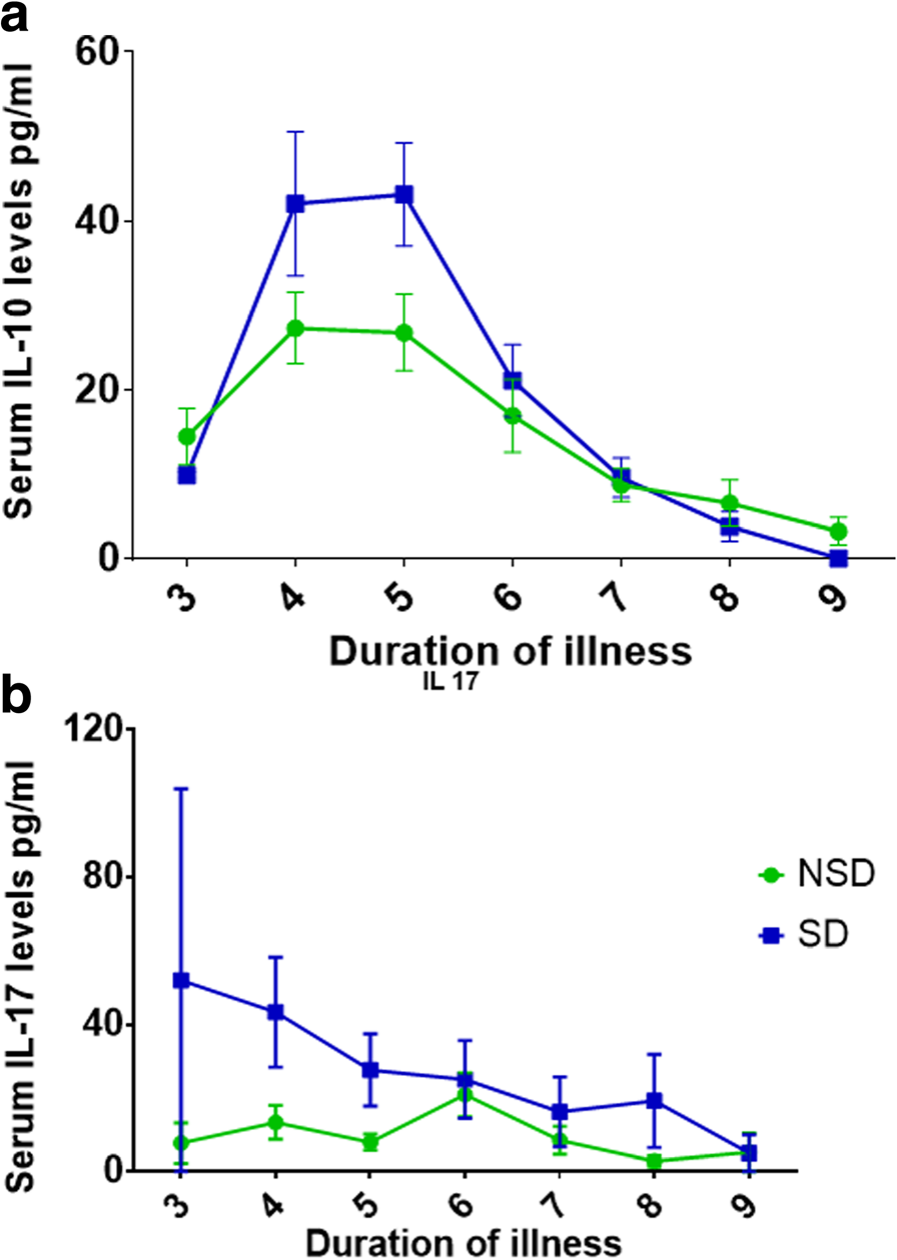
Changes in cytokine levels throughout the course of illness. **a**: Changes in serum IL-10 levels in patients with SD (*n* = 22) and NSD (*n* = 33). The lines indicate the means and standard error of mean (SEM). **b**: Changes in serum IL-17 levels in patients with SD (*n* = 22) and NSD (*n* = 33). The lines indicate the means and standard error of mean (SEM)

Our previous studies and others have shown that elevated IL-10 is associated with severe dengue [30, 31], and therefore, we sought to investigate the changes in IL-10 levels throughout the clinical disease and its association with liver damage. We found that IL-10 levels were elevated in the early course of the illness with IL-10 values being significantly higher on day 5 of illness in patients with SD (*p* = 0.03), when compared to those with NSD (Fig. 4a). As seen in our previous studies, we found that IL-10 inversely correlated with the lowest lymphocyte counts (Spearman’s correlation =0.58, *p* < 0.0001). However, except with being associated with AST levels (Spearman’s correlation =0.14, *p* = 0.04), IL-10 did not associate with any other liver enzyme or clinical parameter.

It has been shown in dengue mouse models that both IL-22 and IL-17 are associated with severe clinical disease and in particularly liver injury [13]. Therefore, we determined the kinetics of changes in IL-17 in acute dengue and its association with liver injury. We found that similar to IL-10, IL-17 was significantly elevated during the early phase of dengue infection. IL-17 levels were significantly elevated in patients with SD on day 4 (*p* = 0.02) and day 5 (*p* = 0.02) when compared to those with NSD (Fig. 4b). IL-17 levels correlated with serum IL-10 levels (Spearmans correlation =0.26, *p* = 0.0002), viral loads (Spearmans correlation = 0.26, *p* = 0.005) and ALT (Spearmans correlation =0.16, *p* = 0.02).

## Discussion

In this study, we have determined the changes in liver enzymes over the course of acute dengue infection and also the relationship of liver involvement with the degree of viraemia, onset and extent of fluid leakage and levels of cytokines. We found that all patients with SD (but who did not have shock) had some degree of liver involvement, while only 15.1 % of those with NSD did not have any liver involvement.

ALT and AST are considered as indicators of liver cell injury as they are released into the circulation following liver cell injury [25]. Although ALT is also found in low concentrations in skeletal muscle, brain and intestinal tissue, it is predominantly considered to be a liver specific enzyme [25]. In contrast, AST is released following damage to liver, cardiac and skeletal muscle [25]. We found that the rise in AST was more prominent than the rise in ALT levels in patients with SD, which probably suggests that other sources apart from the liver could also be contributing to the rise in serum AST levels. Importantly, the mean AST levels rose more than twofold from day 4 to day 6 of onset of illness, and the three patients whose liver transaminases were >1000/IU, had AST levels around 200–250 IU during day 4 of illness. Therefore, determining liver transaminase levels on day 4 or day 5 (day of admission) alone might very much underestimate the level of liver injury during acute dengue infection.

In this study, we also assessed the changes in GGT, ALP, serum bilirubin and serum albumin levels and none of the patients had significant elevations in ALP or serum bilirubin, which suggests that there was minimal cholestasis. Although GGT is also considered to be a marker of cholestasis, it can be elevated due to several other causes including oxidative stress. Oxidative stress has shown to occur in dengue infections, which has been shown to associate with severity of illness [8, 32]. It has been shown that glutathione, which is the main substrate of GGT was reduced in DENV infected cells [33]. Since the GGT levels tend to increase when cellular glutathione levels fall, the increase in GGT levels in dengue could be due to reduction of glutathione levels as a result of oxidative stress [22].

Post mortem studies done in patients who had a fatal outcome have shown that the liver is congested with liver cell necrosis and apoptosis predominantly, in midzonal and centrilobular areas, macrovascular steatosis and councilman bodies, with little inflammation [7, 34, 35]. Centrilobular liver cell necrosis is a typical feature of hypoxic hepatitis, which is the type of liver injury observed in situations of prolonged shock [36]. Although acute liver failure has been reported in many patients with prolonged shock due to dengue [3], acute liver failure has also occurred in the absence of shock [4]. In our cohort of patients with SD, the only 3 patients who had liver transaminases elevated of >1000 IU did not have ultrasound evidence of fluid leakage or any rise in the haematocrit. In addition, 39.4 % of patients with NSD, who did not have any evidence of fluid leakage had more than fourfold rise in their levels of liver transaminases. Therefore, it appears that although hypoxic injury due to reduced hepatic perfusion is probably a cause of liver injury in dengue, other causes such as viral induced liver cell apoptosis and immune mediated damage could also play a role.

Studies done on IL-22 knock out mice have shown that IL-17 plays an important role in the pathogenesis of dengue and also stimulated neutrophil recruitment to the liver, which was found to associate with the severity of liver damage [13]. IL-17 blockade was associated with reduced dengue severity and reduced liver transaminase levels [13]. Although this study shows that IL-17 produced by γδT cells was pathogenic and IL-22 produced by natural killer cells played a protective role, another study in a dengue mouse model showed that early infiltration by NK cells was associated with apoptosis of liver cells [14]. In our cohort of patients, we found that both IL-10 and IL-17 were significantly elevated during early illness, before the patients developed vascular leakage or significant liver injury. Both cytokines were significantly elevated in patients with SD, when compared to NSD, but this difference was not observed after day 5. ALT, which is a liver specific transaminase, was significantly associated with IL-17 levels. Although, our data do not show that IL-17 or IL-10, are in fact responsible for the liver injury in dengue, the findings suggest that it would now be important to investigate their role in more detail in acute dengue infection.

## Conclusion

In summary, our results show that varying degree of acute liver injury are common in dengue viral infections, but did not associate with the degree of viraemia or the onset or extent of fluid leakage. Since the rise in liver transaminase and GGT levels peaked around day 6–7, assays of liver function tests done around day 4–5 (on admission), may significantly underestimate the degree of liver damage. Since both IL-10 and IL-17 were significantly associated with SD, and high levels were seen very early in the illness, their role in the pathogenesis of dengue and dengue liver injury should be further investigated.

## Abbreviations

ALP, alkaline phosphatase; ALT, alanine transaminase; AST, aspartate transaminase; DENV, the dengue virus; GGT, gamma glutamyl transferase; NSD, non severe dengue; PCR, polymerase chain reaction; SD, severe dengue

## Additional files

Additional file 1: Table S1. Number of patients assessed at each time point. (DOCX 11 kb) Additional file 2: Figure S1. Standard curves for determining viral loads. A: standard curve for DENV-1. B: standard curve for DEN-2. C: standard curve for DENV-3. D: standard curve for DENV-4. (TIF 497 kb)
