# Supplementary material for: Patterns and causes of liver involvement in acute dengue infection
Source: BMC Infect Dis. 2016 Jul 8;16:319. doi: 10.1186/s12879-016-1656-2 (PMC4938910; doi:10.1186/s12879-016-1656-2)
Supplement: Additional file 1: Table S1. — Number of patients assessed at each time point. (DOCX 11 kb) [file 12879_2016_1656_MOESM1_ESM.docx]

| **Time point (days of illness)** | **Severe dengue** | **Non severe dengue** |
| --- | --- | --- |
| 3 | 2 | 4 |
| 4 | 8 | 16 |
| 5 | 20 | 31 |
| 6 | 20 | 27 |
| 7 | 20 | 19 |
| 8 | 13 | 11 |
| 9 | 2 | 4 |

**Additional file 1: Table S1: Number of patients assessed at each time point**
