# Supplementary figures and images for: Patterns and causes of liver involvement in acute dengue infection
Source: BMC Infect Dis. 2016 Jul 8;16:319. doi: 10.1186/s12879-016-1656-2 (PMC4938910; doi:10.1186/s12879-016-1656-2)

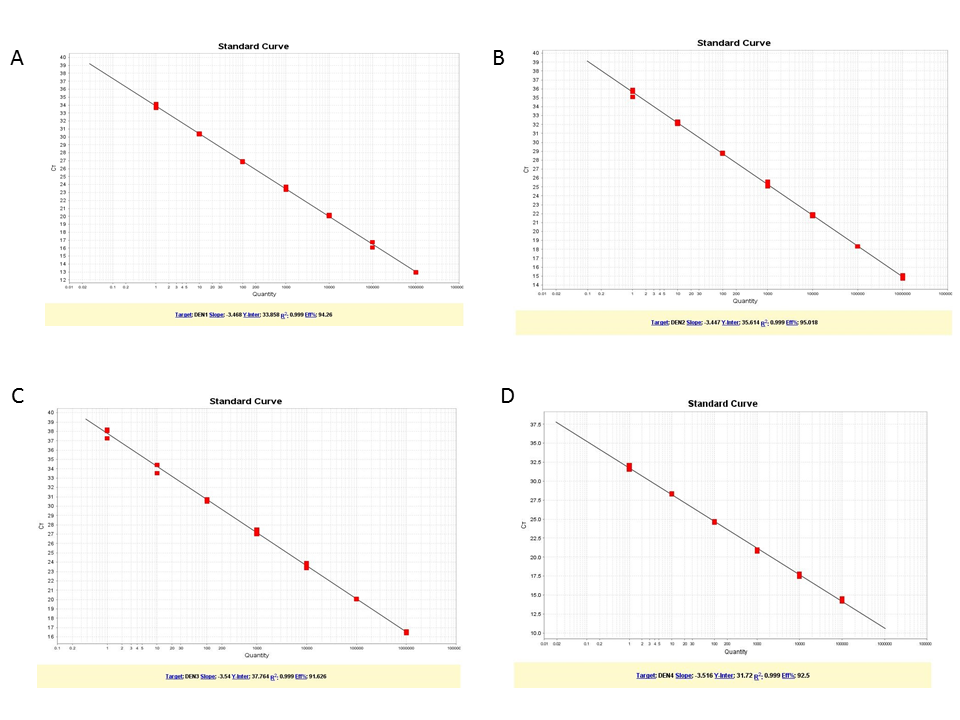

Supplement: Additional file 2: Figure S1. — Standard curves for determining viral loads. A: standard curve for DENV-1. B: standard curve for DEN-2. C: standard curve for DENV-3. D: standard curve for DENV-4. (TIF 497 kb) [file 12879_2016_1656_MOESM2_ESM.tif]
